# Supplementary material for: Yiai Fuzheng decoction inhibits triple-negative breast cancer by remodeling the immune microenvironment
Source: Front Immunol. 2025 Sep 30;16:1615631. doi: 10.3389/fimmu.2025.1615631 (PMC12518410; doi:10.3389/fimmu.2025.1615631)
Supplement: Supplementary file 2 [file DataSheet1.zip › Supplementary File 2/Treatment/C_vs_D_vs_H/06_Pathway_Analysis/Enrichment_Analysis/smpdb_pathway/Enrichment_Barplot.pdf]

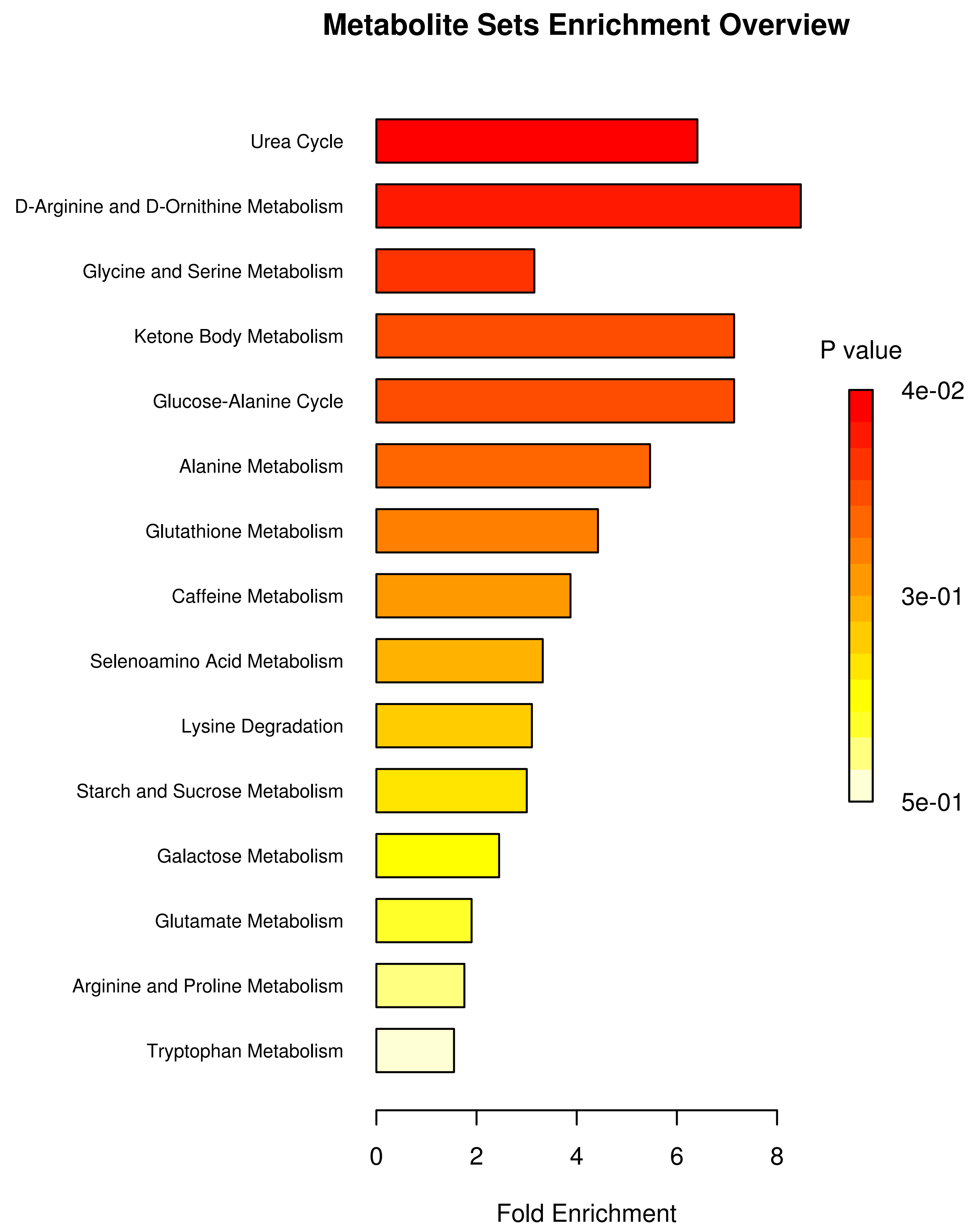

Metabolite Sets Enrichment Overview
Urea Cycle
D-Arginine and D-Ornithine Metabolism
Glycine and Serine Metabolism
Ketone Body Metabolism
Glucose-Alanine Cycle
Alanine Metabolism
Glutathione Metabolism
Caffeine Metabolism
Selenoamino Acid Metabolism
Lysine Degradation
Starch and Sucrose Metabolism
Galactose Metabolism
Glutamate Metabolism
Arginine and Proline Metabolism
Tryptophan Metabolism
P value
4e-02
3e-01
5e-01
0
2
4
6
8
Fold Enrichment
